# Oxidative Score and Microvesicle Profile Suggest Cardiovascular Risk in Chronic Kidney Disease

**DOI:** 10.3390/antiox14020178

**Published:** 2025-02-03

**Authors:** Gemma Valera-Arévalo, María del Mar Rodríguez-San Pedro, Paula Jara Caro, Víctor Cabanillas, María Gabriela Ortiz-Diaz, Andrea Figuer, Claudia Yuste, Rafael Ramírez, Matilde Alique, Enrique Morales, Natalia Guerra-Pérez, Julia Carracedo

**Affiliations:** 1 Department of Genetics, Physiology and Microbiology (Unit of Animal Physiology), Faculty of Biology, Universidad Complutense de Madrid, 28040 Madrid, Spain; mararodr@ucm.es (M.d.M.R.-S.P.); vicabani@ucm.es (V.C.); maorti11@ucm.es (M.G.O.-D.); natalgue@ucm.es (N.G.-P.); 2 Instituto de Investigación Sanitaria Hospital 12 de Octubre (imas12), RICORS 2040, 28041 Madrid, Spain; paulajara.caro@salud.madrid.org (P.J.C.); claudia.yuste@salud.madrid.org (C.Y.); emoralesr@senefro.org (E.M.); 3 Department of Nephrology, Hospital Universitario 12 de Octubre, RICORS 2040, 28041 Madrid, Spain; 4 Department of Systems Biology, Universidad de Alcalá, 28871 Alcalá de Henares, Spain; andrea.figuer@edu.uah.es (A.F.); manuel.ramirez@uah.es (R.R.); matilde.alique@uah.es (M.A.); 5 Instituto Ramón y Cajal Ramón y Cajal de Investigación Sanitaria (IRYCIS), 28034 Madrid, Spain

**Keywords:** chronic kidney disease, oxidative stress, microvesicles, cardiovascular risk, endothelial dysfunction, hemodialysis, peritoneal dialysis, reactive oxygen species, antioxidants, coagulation

## Abstract

Chronic kidney disease (CKD) is associated with a high incidence of cardiovascular disease (CVD) due to the accumulation of uremic toxins, altered redox state, and chronic systemic inflammation. This study aimed to analyze the relationship between the redox status of patients with CKD and the phenotype of microvesicles (MVs) subtypes, and cardiovascular events. The oxidative stress level of each participant was determined using an individualized OXY-SCORE. The relationship between pro-oxidant and antioxidant parameters and the expression of membrane markers in endothelial-derived microvesicles (EMVs) and platelet-derived microvesicles (PMVs) was established. Patients with advanced CKD (ACKD) and hemodialysis (HD) had a higher OXY-SCORE than healthy subjects (HS), whereas peritoneal dialysis (PD) patients had similar scores to HS. PD patients showed elevated PMVs and CD41 expression, whereas HD patients had higher EMVs and CD31 expression. Patients with ACKD had higher tissue factor (TF) expression in the PMVs and EMVs. TF expression was correlated with xanthine oxidase (XO) activity and was negatively correlated with antioxidant parameters. Patients with cardiovascular events show elevated TF. In conclusion, microvesicles and oxidative stress may serve as markers of cardiovascular risk in CKD, with TF expression in PMVs and EMVs being potential predictive and prognostic biomarkers of CVD.

## 1. Introduction

Chronic kidney disease (CKD) has a worldwide prevalence of 9% and is characterized by progressive loss of kidney function over a period of at least three months [1]. CKD can have different causes, although the most common are hypertension (HTN) and diabetes mellitus (DM) [2]. CKD has different stages depending on the glomerular filtration rate (GFR), with stages 4 and 5 being known as advanced chronic kidney disease (ACKD) (GFR < 15 mL/min) [3]. The treatment of CKD consists of blood purification using dialysis techniques. Within dialysis, hemodialysis (HD) consists of extracorporeal filtration of blood through a dialyzer and is considered a less biocompatible technique. On the other hand, peritoneal dialysis (PD) uses the patient’s peritoneal membrane as a filtering membrane [4].

The main cause of mortality in CKD is cardiovascular disease (CVD), preceded by underlying multifactorial endothelial dysfunction, accompanied by an altered REDOX state as well as alterations in factors involved in coagulation processes that contribute to increased thrombotic and hemorrhagic risk [5].

REDOX imbalance is characterized by an accumulation of reactive oxygen species (ROS), increased activity of pro-oxidant enzymes such as xanthine oxidase (XO), increased levels of lipid peroxidation products such as malondialdehyde (MDA), measured as thiobarbituric acid reactive substances or TBARS in plasma, and decreased activity of antioxidant systems such as catalase (CAT) and superoxide dismutase (SOD) enzymes [6]. Oxidative stress is a consequence of CKD and contributes to disease progression. It occurs in the early stages of CKD and can be assessed as an early indicator of CKD [2]. An altered REDOX state has been observed in other inflammatory diseases, such as arthritis, in whose pathophysiology plays a key role [7].

In addition, HD predisposes to an accumulation of pro-oxidant species due to the filtration of low molecular weight antioxidants, the use of anticoagulants such as heparin or the use of non-biocompatible membranes in the filtration system (cellulose, polysulphones, etc.) [8]. Previous studies have shown that patients undergoing renal replacement therapy such as HD or PD have higher levels of pro-oxidant enzymes in the blood/plasma than those with non-dialysis-dependent CKD in the ACKD stage [9].

Endothelial dysfunction, which consists of the loss of endothelial permeability as well as the assumption of a secretory senescent phenotype (SASP) by endothelial cells, has been postulated as another precursor of CVD and acute cardiovascular events [10]. Endothelial dysfunction is also closely related to CKD and endothelial cell senescence is also characterized by increased resistance to apoptosis, increased release of ROS release and increased expression of adhesion molecules on endothelial cells, such as CD31 (PECAM-1), as well as the production of thrombotic factors, such as tissue factor (TF or CD142), which promotes fibrin formation by regulating the extrinsic coagulation system, allowing clot stabilization in the presence of platelets [11].

One factor to consider is the release of extracellular vesicles (EVs). EVs constitute an intercellular communication system because of the microRNAs contained inside them, which modulate protein synthesis and the expression of different molecules on their surface. Both their content and the molecules expressed depend on the cells that release them [12]. The release of EVs has been studied in different pathologies as potential biomarkers or therapeutic targets. Cancer is an important area of research on EVs, and the content of these EVs has been studied, showing specific microRNAs depending on the type of cancer (miR-193a in colon cancer, miR-335 in hepatocellular cancer and miR-155 in melanoma) [12]. Within the EVs there are different groups depending on their size. Microvesicles (MVs) have a size of 100–1000 nm and exosomes have a size of approximately 30–150 nm [12]. Interestingly, endothelial dysfunction has also been associated with an increased release of endothelium-derived MVs (EMVs) in patients with heart failure, which may be useful as a marker of endothelial damage [12]. EMVs with membrane molecules such as CD31 (PECAM-1, endothelial cells and platelet adhesion molecules) act as signalers/mediators of endothelial dysfunction and impaired endothelial permeability, promoting coagulation and inflammation [13].

In patients with CKD, platelets undergo a process of hyperactivation due to the accumulation of uremic toxins and the state of chronic systemic inflammation experienced by these patients. This hyperactivation is characterized by the increased expression of CD41 (platelet GPIIb; integrin IIb, promoter of platelet activation) or TF [14]. Owing to this activation, an increased release of platelet-derived MVs (PMVs) has been observed in patients with uremia and DM patients [15].

The aim of this study was to analyze the connection between the REDOX status of CKD patients and the phenotype of EVs subtypes of different origins, as well as to study their possible relationship with cardiovascular events. To this end, the oxidative stress of each patient or healthy subject was determined in depth using an individualized SCORE of the REDOX status. In addition, the relationship between different pro-oxidant and antioxidant parameters and the degree of expression of membrane markers in EMVs and PMVs, such as CD31, CD41, and TF, as well as their relationship with different renal replacement therapies and antiplatelet treatment agents, was established.

## 2. Materials and Methods

### 2.1. Healthy and Chronic Kidney Disease Sample Donors

**Figure 1:**
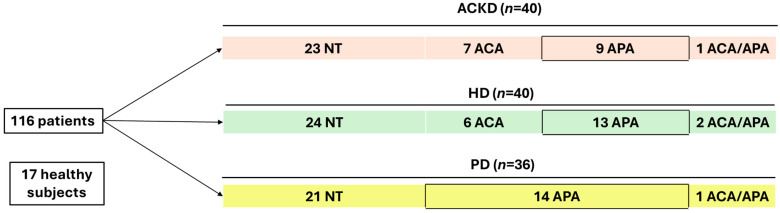
Study design. ACKD, advanced chronic kidney disease; HD, hemodialysis; PD, peritoneal dialysis; NT, non-treatment with anticoagulant or antiplatelet agents; APA, antiplatelet agents; ACA, anticoagulant agents.

This cross-sectional study included 116 patients with CKD (40 with advanced chronic kidney disease (ACKD), 40 on hemodialysis (HD), and 36 on peritoneal dialysis (PD)). A total of 17 healthy subjects (HS) were included to establish the standard values for the parameters studied. Data were collected from patients treated with anticoagulant and antiplatelet drugs, considering their possible effect on the variables analyzed. The study design is illustrated in Figure 1.

Subjects included in the HD and PD groups were maintained in the same clinical condition for at least 6 months before being recruited for the study. Patients with neoplasms, infections, and active autoimmune or inflammatory diseases were excluded. All the patients were recruited from the Nephrology Department of the Hospital Universitario 12 de Octubre, Madrid (Spain). All procedures were performed in accordance with the Declaration of Helsinki of the World Medical Association and the protocol was approved by the Ethics Committee of the Research Institute of Hospital 12 de Octubre (Approval nº 17/407). Written informed consent was obtained from all the participants before recruitment. The clinical variables of the study participants are listed in Table 1.

### 2.2. Blood Collection and Preparation

Peripheral blood samples were collected from each subject by venous puncture in EDTA-anticoagulated vacutainer tubes during routine examinations of patients at the Nephrology Department of the Hospital Universitario 12 de Octubre, Madrid (Spain). Subsequently, the samples were transported to the Animal Physiology Unit of the Faculty of Biology of Complutense University of Madrid (Spain). Within 24 h of collection the blood was divided into several portions to obtain samples for different experimental assays, and, in addition, 1 or 2 aliquots were stored at −80 °C for later determination.

Platelet-free plasma was isolated from the peripheral whole blood of each patient or HS by centrifugation (1250× *g*, 20 min) (Eppendorf Benchtop Refrigerated centrifuge 5403 with rotor 16F24-11, Eppendorf, Hamburg, Germany) and stored at −80 °C in aliquots for further processing for oxidative stress and MVs flow cytometry studies.

### 2.3. Leukocytes Density Gradient Separation

Isolation of polymorphonuclear leukocytes (PMN) and mononuclear leukocytes (MN) was performed using the Ficoll gradient technique. At the time of blood processing, the samples were transferred to a 50 mL tube, mixed with an equal volume of phosphate-buffered saline (PBS) and then underlaid with Histopaque^®^ 1.119 g/mL and 1.077 g/mL (Sigma-Aldrich, Madrid, Spain) before centrifugation at 800× *g* for 30 min with brake off (Eppendorf Benchtop Refrigerated centrifuge 5403 with rotor 16F24-11, Eppendorf, Hamburg, Germany). Leukocyte subtypes were then transferred to fresh tubes and washed three times with PBS (1250× *g*, 20 min) before being distributed into several aliquots at a concentration of 1 × 10^6^ cells/mL.

### 2.4. Oxidative Stress Parameters

Oxidative stress parameters were studied in plasma, as well as in PMN and MN.

#### 2.4.1. Xanthine Oxidoreductase Activity

Xanthine oxidase (XO) activity was quantified in plasma (40 μL), PMN and MN (1 × 10^6^ cells/mL) using the Amplex^®^ Red Xanthine/Xanthine Oxidase Assay Kit (A-22182 Amplex Red Xanthine/Xanthine Oxidase Assay Kit, Molecular Probes, Paisley, UK) [16]. Before use, the PMN and MN were resuspended in a lysis buffer and centrifuged (10,000× *g*, at 4 °C, 20 min) (Eppendorf Benchtop Refrigerated centrifuge 5403 with rotor 16F24-11, Eppendorf, Hamburg, Germany) to obtain the intracellular soluble fraction. Briefly, XO catalyzes the oxidation of hypoxanthine to uric acid and superoxide. Subsequently, superoxide spontaneously degrades to hydrogen peroxide (H_2_O_2_) and, in the presence of horseradish peroxide, reacts with the Amplex^®^ Red reagent to render resofurin, the absorbance of which was measured at 560 nm. XO activity is expressed as mU XO/mg or mU/mL.

#### 2.4.2. Glutathione Peroxidase Activity

Glutathione peroxidase (GPx) activity was determined in plasma (10 μL), PMNs and MNs (1 × 10^6^ cells/mL) by colorimetric assay using the EnzyChrom™ Glutathione Peroxidase Assay Kit EGPX-100 (BioAssay Systems, Hayward, CA, USA), according to the protocol provided [16]. The procedure quantifies the decrease in absorbance, which is directly proportional to the NADPH-based GPX enzyme activity. Before use, PMN and MN were resuspended in a lysis buffer, sonicated, and centrifuged (11,000× *g*, at 4 °C, 10 min) (Eppendorf Benchtop Refrigerated centrifuge 5403 with rotor 16F24-11, Eppendorf, Hamburg, Germany) to obtain the intracellular soluble fraction. For this determination, 10 μL of plasma and 10 μL of the intracellular soluble fraction of PMNs and MNs were used, and absorbance was monitored at 340 nm for 4 min (t0; t4). Activity is expressed as units (U) of GPx/mg protein or U/L.

#### 2.4.3. Lipid Peroxidation Assay

Lipid peroxidation was determined using the thiobarbituric acid reactive substance (TBA) assay, which measures malondialdehyde (MDA) as a product of lipid peroxidation at 531 nm. This procedure used 300 μL of plasma, and PMN and MN (1 × 10^6^ cells/mL) using the MDA Assay Kit (BioVision Inc., Milpitas, CA, USA) [16]. Lipid peroxidation was calculated using linear regression derived from an MDA standard curve and expressed as nmol MDA/mg protein or TBARS/mL.

#### 2.4.4. Glutathione Content Assay

For glutathione analysis, PMN and MN (1 × 10^6^ cells/mL) were homogenized in a lysis buffer, resuspended by sonication for 10 s (three times), centrifuged at 11,000× *g* for 10 min at 4 °C (Eppendorf Benchtop Refrigerated centrifuge 5403 with rotor 16F24-11, Eppendorf, Hamburg, Germany), and supernatants were collected as intracellular fraction Oxidized (GSSG) and reduced (GSH) glutathione were quantified from the reaction with o-phthalaldehyde (OPT) (G4251-5G, G4376-500 MG, 79760-5G, respectively, Sigma Aldrich, Spain) in a pH range of 8–12 according to the glutathione status, resulting in a fluorescent product measured at 420 nm. GSH and GSSG concentrations were expressed in nmol/mg protein or nmol/mL, which allowed the calculation of the glutathione GSH/GSSG REDOX ratio.

#### 2.4.5. Catalase Activity

Extracellular quantification of H_2_O_2_ was determined as an indirect measure of catalase activity (CAT), an enzyme that reacts with hydrogen peroxide (H_2_O_2_) to produce water and oxygen. PMN and MN (1 × 10^6^ cells/mL) were resuspended in a lysis solution, sonicated, and centrifuged (3200× *g*, 20 min, 4 °C) to obtain clear supernatants. According to the fluorometric kit protocol (A-22180 Amplex^®^ Red Catalase Assay Kit, Molecular Probes, Paisley, UK), 25 µL of PMN and MN supernatants and directly plasma (25 µL) were then mixed with 40 µM H_2_O_2_ and incubated in the dark for 30 min at room temperature. Samples were incubated for 30 min at 37 °C with the working solution containing horseradish peroxidase (HRP) reacting with any unreacted H_2_O_2_ (1:1) to yield fluorescent resorufin, which was measured by excitation-emission detection at 530–590 nm. The results were expressed as U CAT/mg protein or U/mL.

#### 2.4.6. Superoxide Dismutase Activity

Superoxide dismutase (SOD) activity was determined by a spectrophotometric assay using an EnzyChromTM ESOD-100 kit (BioAssay Systems, Hayward, CA, USA). SOD inhibits the reduction of WST-1 (water-soluble tetrazolium) salt by XO (xanthine/xanthine oxidase) and water-soluble formazan production. Plasma (20 µL), and PMN and MN (1 × 10^6^ cells/mL) supernatants (20 µL) were collected by centrifugation (1500× *g*, 10 min; 4 °C) (Eppendorf Benchtop Refrigerated centrifuge 5403 with rotor 16F24-11, Eppendorf, Hamburg, Germany) after sonication with cold lysis solution and were analyzed according to the manufacturer’s protocol. The absorbance of formazan reached its maximum at 438–460 nm^16^. The results were expressed as units (U) of SOD/mg protein or U SOD/mL.

#### 2.4.7. Protein Content Assay

The protein content of PMNs and MNs was determined using the bicinchoninic acid (BCA) protein assay kit protocol (Sigma-Aldrich, Madrid, Spain), according to the manufacturer’s instructions [16].

### 2.5. OXY-SCORE Index Determination

The study of patients’ oxidative status was completed by calculating the OXY-SCORE proposed by Veglia et al. (2010) [17], which encompasses the different parameters analyzed in our study. The score evaluates the redox status of each patient individually based on oxidative stress related to pro-oxidant and antioxidant factors. First, a logarithmic transformation of the parameters that did not follow a normal distribution was performed, favoring a more symmetrical distribution. The variables were standardized by applying the formula: Zij = (xij − mj)/sj, where Zij (standardized value of variable j for subject); xij (raw measure (possibly log transformed) of variable j for subject i); mj (mean of variable j); sj (standard deviation of variable j) are related. Subsequently, the individual scores were added to obtain the overall score for each patient and healthy subject.

### 2.6. Circulating Microvesicles Isolation

MVs were determined from platelet-free plasma (1 mL) within the first 4 h after blood collection by a two-step centrifugation protocol and from each patient or HS in the study. Centrifugation at 11,000× *g* for 35 min at 20 °C was performed to obtain a pellet with isolated MVs.

### 2.7. Phenotyping of Microvesicles by Flow Cytometry

**Figure A1:**
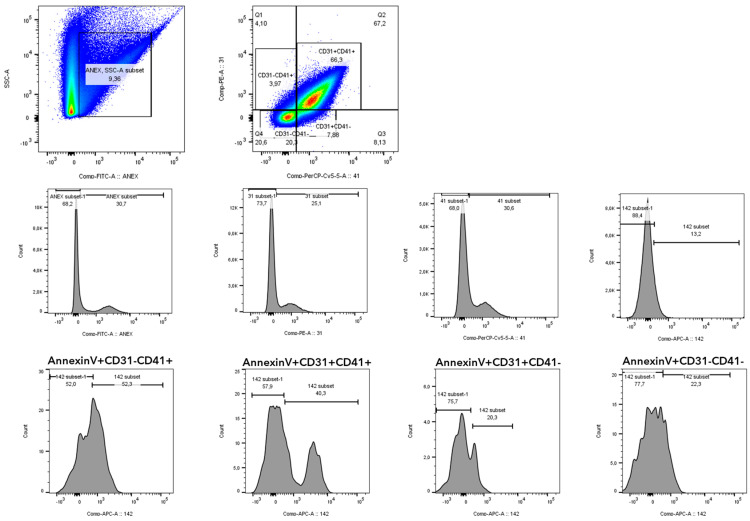
Representative flow citometry findings of MVs subsets and CD142 expression.

After isolation, MVs were phenotyped according to the International Society for Extracellular Vesicles (ISEV2023) guidelines (MISEV2023) [18]. The total number of AnnexinV+MVs, Annexin V+CD31+CD41–MVs (EMVs), AnnexinV+CD31+CD41+MVs (PMVs), as well as CD142+ tissue factor (TF) expression CD142+ on EMVs and PMVs, were determined as previously described [4]. Optimal fluorescence settings for defining microparticles and for MVs analysis were previously measured using the SpheroTM Nanofluorescent Particle Size Standard (Cat. no. NFPPS-52-4K Spherotech, Lake Forest, IL, USA) compound by blank beads and four bead sizes: 0.22, 0.45, 0.88 and 1.32 µm. One drop of calibration bead set solution was diluted in 500 μL of filtered phosphate-buffered saline (PBS) and acquired and analyzed following the manufacturer’s requirements. A quadruple-staining immunofluorescence technique was used, and nanoscale flow cytometry analysis was performed. Fluorochromes-conjugated monoclonal antibodies (mAbs) conjugated against Annexin V (Annexin V-FITC Kit; Miltenyi Biotec, Bergisch Gladbach, Germany), CD41/integrin subunit alpha 2b (MEM-06 clone, peridnine chlorophyll protein, PerCP; Invitrogen, Carlsbad, CA, USA), CD31/platelet and endothelial cell adhesion molecule 1 (PECAM1) (WM-59 clone, phycoerythrin, PE; BD Biosciences, San Jose, CA, USA), and CD142/tissue factor (HTF-1 clone, allophycocyanin, APC; Invitrogen) were used in a surface-staining panel with appropriate isotype controls. Briefly, platelet-free plasma samples were centrifuged at 11,000× *g* for 20 min to isolate MVs and resuspended in Annexin V-binding buffer (Annexin V-FITC Kit; Miltenyi Biotec). Samples were incubated with mAbs for 45 min at room temperature in the dark, fixed with Cell Fix (BD Biosciences), and stored at 4 °C until assessment. A sample without mAb or Annexin-V was used as a flow cytometry control sample, and a sample without mAb (but with Annexin-V) was used to establish the gate for positive Annexin-V events, whose high affinity for the Phosphatidylserine, exposed in the membrane of MVs, phenotypes these elements. At least 200,000 events were acquired using a FACSaria cytometer (BD Biosciences), and FlowJo-V10 (Tree Star, Ashland, OR, USA) was used to analyze the data with the support of the cytometry-associated research center (Cytometry and Fluorescence Microscopy Unit (CCMF)) at the Complutense University of Madrid (Spain). The gating strategy is shown in Figure A1. The mean fluorescence intensities (MFI) of the expression of each marker were explored, as well as the percentages of different MVs.

### 2.8. Statistical Analysis

Data are represented as n (percentage), and mean fluorescence intensity (MFI) and are expressed as mean ± standard deviation (SD). Data sets were checked for normality for each variable using the Kolmogorov–Smirnov test and then analyzed with one-way analysis of variance (ANOVA) followed by Tukey’s post-hoc test. Kruskal–Wallis test was used for data that did not follow a normal distribution. The chi-square test was used to analyze the clinical variables. The correlation between oxidative stress parameters and TF expression in EMVs and PMVs was analyzed using Spearman’s correlation, and the *p*-value from the Spearman rank coefficient analysis was used to determine significance. Statistical analyses and graphical representation were performed using SPSS 21.0 (Armonk, NY, USA) and Graphpad Prism 8.02 (GraphPad InStat Inc., San Diego, CA, USA) software. The level of statistical significance was set at *p* = 0.05.

## 3. Results

The clinical characteristics of the patients with CKD and HS are shown in Table 1. There were no significant differences in age or sex between the study groups. Subjects with ACKD, HD and PD had a higher incidence of hypertension than those with HS (87%, 76%, and 90%, respectively, vs. 6%). Patients with ACKD had a higher incidence of diabetes mellitus than those with healthy patients (HS) (47% vs. 12%). In ACKD, HD and PD, the incidence of dyslipidemia was 81%, 48% and 64% and that of hyperuricemia was 65%, 24% and 55%, respectively. None of the HS had any of these conditions. No significant differences were observed in the number of smokers between the different study groups. The incidence of cardiovascular events was higher in patients with ACKD, HD and PD (69%, 75% and 67%, respectively) compared to HS. The percentage of ACKD, HD and PD patients treated with erythropoietin was 53%, 100% and 55%, respectively. Statin therapy was administered to 78%, 27%, and 58% of ACKD, HD, and PD patients, respectively. The proportion of ACKD, HD, and PD patients treated with allopurinol was 56%, 21%, and 52%, respectively. The percentage of subjects taking antiplatelet drugs was 17.5% in ACKD, 25% in HD and 19.4% in PD, respectively. The percentage of subjects taking anticoagulant drugs was 20%, 12.5% and 2% respectively. HS subjects did not undergo any of these treatments.

**Figure 2:**
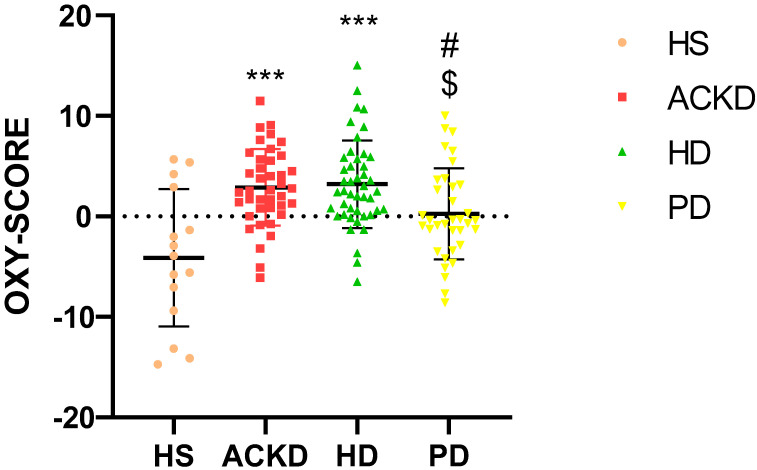
Comparison of oxidative state (OXY-SCORE) between CKD groups analyzed and treatments. *** *p* < 0.001 vs. HS; ^#^ *p* < 0.05 vs. ACKD; ^$^ *p* < 0.05 vs. HD (Kruskal–Wallis). HS, healthy subjects; ACKD, advanced chronic kidney disease; HD, hemodialysis; PD, peritoneal dialysis.

Differences in the oxidative status of participants in the different groups were studied using the OXY-SCORE described in the methodology, and higher oxidative stress was observed in patients with ACKD and HD compared with HS. Patients on PD had similar oxidative stress scores to HS (Figure 2). The results for the individual pro-oxidant and antioxidant parameters studied in plasma, PMN and MN are shown in Table A1 and Table A2.

**Figure 3:**
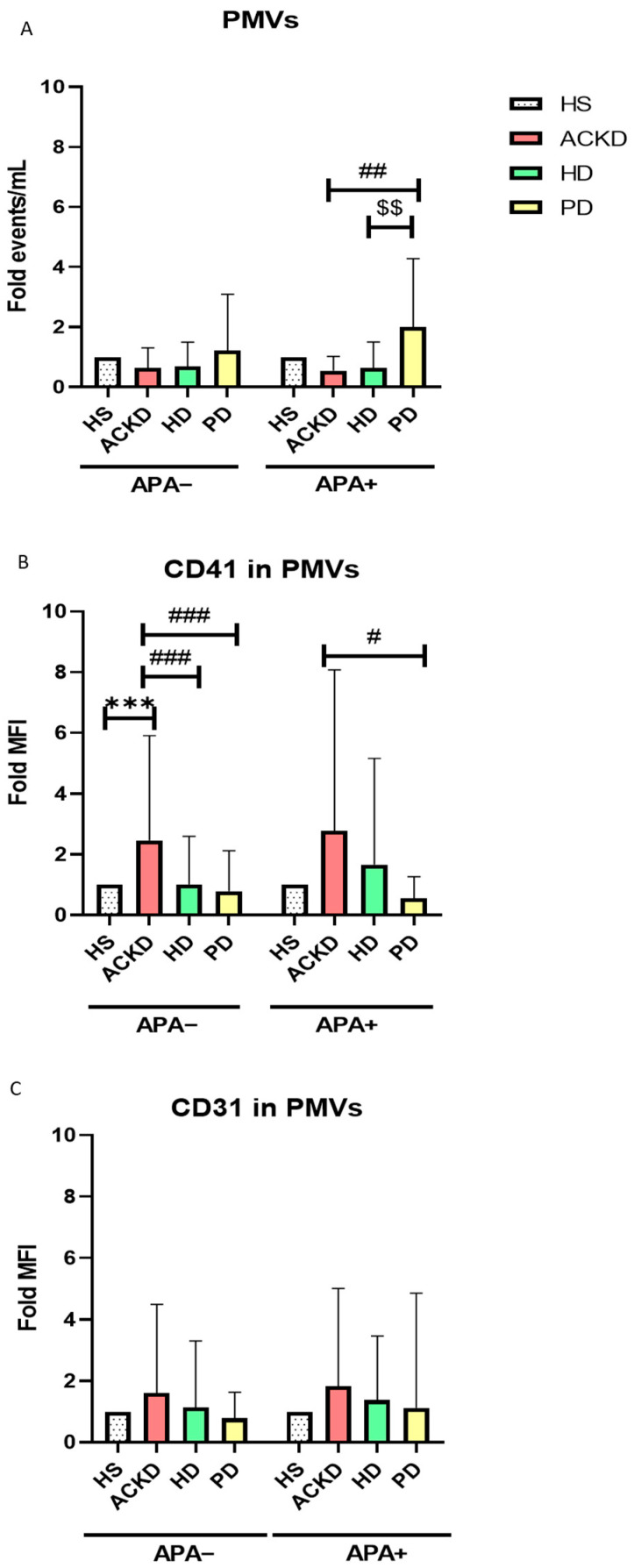
Expression of PMVs, CD31, and CD41. Levels of PMVs (**A**), as well as CD31 (**B**) and CD41 (**C**) expression were expressed as relative change (Fold) compared to HS in patients without antiplatelet agents (APA−) and treated with antiplatelet agents (APA+). ^#^ *p* < 0.05 vs. ACKD; ^##^ *p* < 0.01 vs ACKD; ^###^ *p* < 0.001 vs. ACKD; ^$$^ *p* < 0.005 vs. ACKD (ANOVA and Kruskal–Wallis); *** *p* < 0.001. PMVs, platelet microvesicles; HS, healthy subjects; ACKD, advanced chronic kidney disease; HD, hemodialysis; PD, peritoneal dialysis; MFI, mean fluorescence intensity; APA, antiplatelet agents. Sample size: 40 ACKD, 40 HD, 36 PD and 17 HS.

Plasma levels of PMVs, as well as CD31 and CD41 expression in PMVs were evaluated. The study was performed separately for patients who were treated with APA drugs or without APA or anticoagulant therapy, as well as using the relative change (Fold) in all groups to establish changes with respect to HS. Patients in PD and APA treatment had elevated plasma levels of PMVs compared to patients with ACKD and HD patients with APA therapy (Figure 3A). CD41 and CD31 expression in PMVs was higher in the ACKD group not treated with APA than HS, HD and PD not treated with APA (Figure 3B,C), although a trend was observed for CD31. PD patients treated with APA showed lower levels of CD41 expression in PMVs compared to ACKD treated with APA. Treatment with APA did not seem to affect these parameters. Total number and phenotype of PMVs is shown in Table A3.

**Figure 4:**
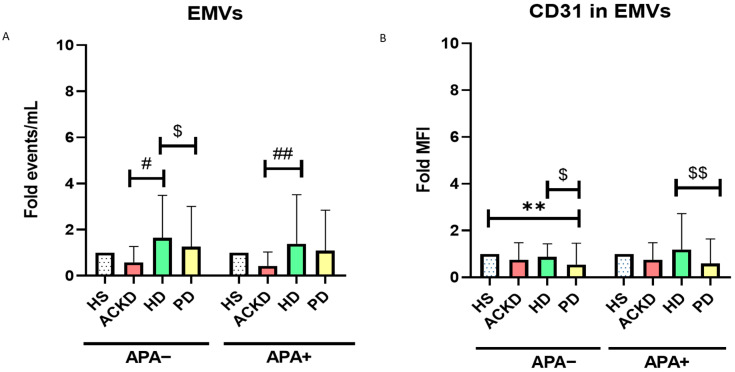
Expression of EMVs, CD31 and CD41. Levels of EMVs (**A**) and CD31 expression in EMVs (**B**) were expressed as relative change (Fold) compared to HS in patients without antiplatelet agents (APA−) and treated with antiplatelet agents (APA+). ** *p* < 0.05 vs HS; ^#^ *p* < 0.05 vs. ACKD; ^##^ *p* < 0.01 vs. ACKD; ^$^ *p* < 0.05 vs. HD; ^$$^ *p* < 0.01 vs. HD (ANOVA). EMVs, endothelial microvesicles; HS, healthy subjects; ACKD, advanced chronic kidney disease; HD, hemodialysis; PD, peritoneal dialysis; MFI, mean fluorescence intensity; APA, antiplatelet agents. Sample size: 40 ACKD, 40 HD, 36 PD and 17 HS.

After studying the release of PMVs, we studied the release of EMVs into plasma. The study was performed separately for patients according to treatment with APA drugs or the absence of APA or anticoagulant treatment, as well as using the relative change (Fold) in all groups with respect to HS. The number of EMVs was higher in HD subjects with or without APA therapy compared to ACKD with or without APA therapy. Moreover, PD patients without APA therapy showed lower levels of EMVs compared with HD patients without APA therapy (Figure 4A). CD31 expression in EMVs was lower in PD patients without APA therapy compared to HS, ACKD and HD patients without APA therapy. Moreover, PD patients undergoing APA therapy showed lower expression of CD31 in EMVs compared to HD patients treated with APA (Figure 4B). APA treatment did not seem to have an effect on these parameters. The total number and phenotype of EMVs is shown in Table A3.

**Figure 5:**
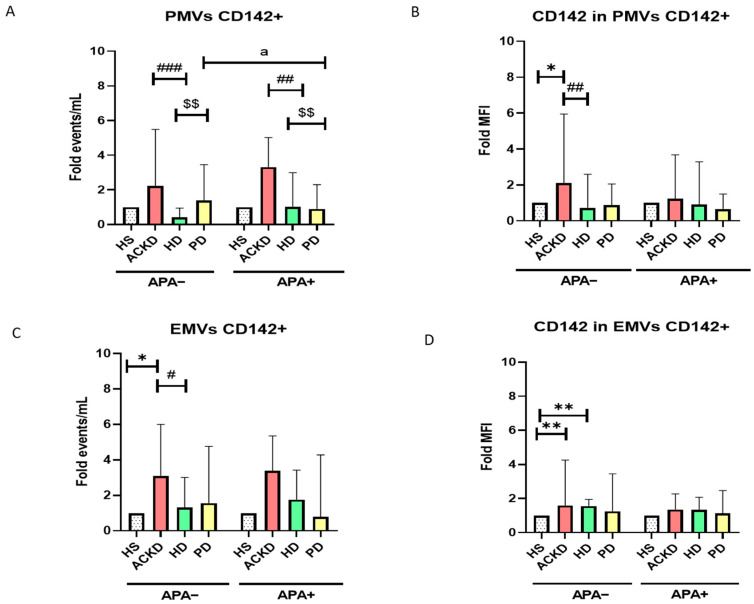
CD142 expression in PMV and EMV. Levels of PMV CD142+ (**A**), MFI of CD142 expression in PMV (**B**), total number of EMVs CD142+ (**C**) and MFI of CD142 expression in EMVs (**D**) were expressed as a relative change (Fold) compared to HS in patients without antiplatelet agents (APA-) and treated with antiplatelet agents (APA+). * *p* < 0.05 vs. HS; ** *p* < 0.01 vs. CT; ^#^ *p* < 0.05 vs. ACKD; ^##^ *p* < 0.01 vs. ACKD; ^###^ *p* < 0.001 vs. ACKD; ^$$^ *p* < 0.01 vs. HD; ^a^ *p* < 0.05 vs. DP (APA−) (ANOVA and Kruskal–Wallis). PMVs, platelet microvesicles; EMVs, endothelial microvesicles; HS, healthy subjects; ACKD, advanced chronic kidney disease; HD, hemodialysis; PD, peritoneal dialysis; MFI, mean fluorescence intensity; APA, antiplatelet agents. Sample size: 40 ACKD, 40 HD, 36 PD and 17 HS.

To evaluate the procoagulant activity of MVs, CD142 (TF) expression in PMVs and EMVs was analyzed. The study was carried out separately for patients according to treatment with APA drugs or the absence of APA or anticoagulant treatment, as well as using the relative change (Fold) in all groups with respect to HS TF expression was studied as a function of the number of MVs expressing TF as well as the amount of TF expressed by MVs (directly proportional to the MFI). The number of PMVs TF+ was lower in HD patients treated or not treated with APA therapy compared to ACKD patients treated or not treated with APA therapy. The number of PMVs TF+ was higher in PD patients treated or not treated with APA therapy compared to HD patients treated or not treated with APA therapy. Levels of PMVs TF+ were lower in PD patients with APA therapy compared to PD patients without APA therapy (Figure 5A). Levels of EMVs TF+ were higher in ACKD patients not treated with APA compared to HS. Moreover, the levels of EMVs TF+ in HD patients not treated with APA were lower compared to ACKD patients not treated with APA (Figure 5C). Expression of TF in PMVs in HD patients not treated with APA was lower compared to HS and ACKD patients not treated with APA (Figure 5B). Expression of TF in EMVs was higher in ACKD and HD patients treated with APA compared to HS (Figure 5D). The total number of PMVs CD142+ and EMVs CD142+ as well as the expression of CD142 per MVs (MFI) is shown in Table A3.

**Figure 6:**
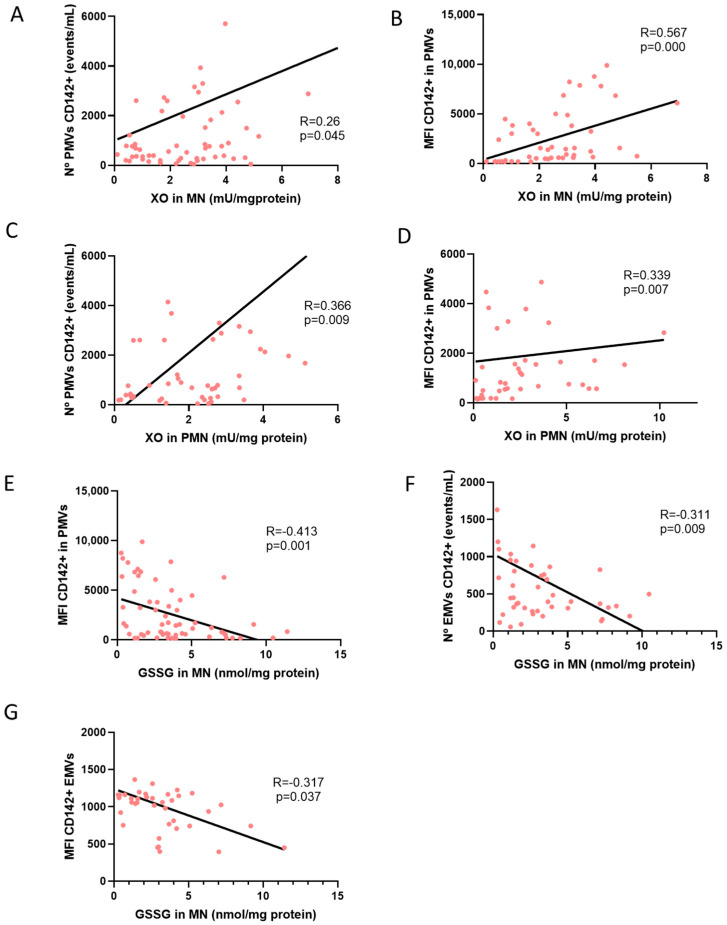
Pro-oxidant activity and TF expression in MV. Correlation between XO in MN and the total number of CD142+PMVs (**A**) and MFI of CD142 expression in PMVs (**B**). Correlation between XO in PMN and total number of CD142+PMVs (**C**) and MFI of CD142 expression on PMVs (**D**). Correlation between GSSG in MN and MFI of CD142 expression (**E**). Correlation between GSSG in MN and the total number of CD142+EMVs (**F**). Correlation between GSSG in MN and MFI of CD142 expression in EMVs (**G**) (Spearman correlation). XO, xanthine oxidase; GSSG, glutathione disulfide; MN, mononuclear leukocytes; PMN, polymorphonuclear leukocytes; PMVs, platelet microvesicles; MFI, mean fluorescence intensity; EMVs, endothelial microvesicles. The total number of ACKD, HD and PD patients with or without APA therapy were analyzed. Sample size: 40 ACKD, 40 HD, 36 PD and 17 HS).

Correlations between pro-oxidant parameters and TF (CD142+) expression in EMVs and PMVs are shown in Figure 6. ACKD, HD and PD patients with or without APA therapy were analyzed. The number of PMVs expressing TF and the expression (MFI) of TF in PMVs showed a positive correlation with XO activity in MN (r = 0.26, *p* = 0.045 and r = 0.567, *p* = 0.000; Figure 6A,B). The number of PMVs expressing TF and the expression (MFI) of TF in PMVs showed a positive correlation with XO in PMN (r = 0.336, *p* = 0.000 and r = 0.339, *p* = 0.000; Figure 6C,D). The expression (MFI) of TF in PMVs, the number of EMVs expressing TF and the expression (MFI) of TF in EMVs showed a negative correlation with the amount of GSSG in MN (r = −0.413, *p* = 0.000; r = −0.31, *p* = 0.009 and r = −0.317, *p* = 0.037; Figure 6E–G). The remaining correlations between pro-oxidant parameters and TF expression in EMVs and PMVs are shown in Table A4 and Table A5.

**Figure 7:**
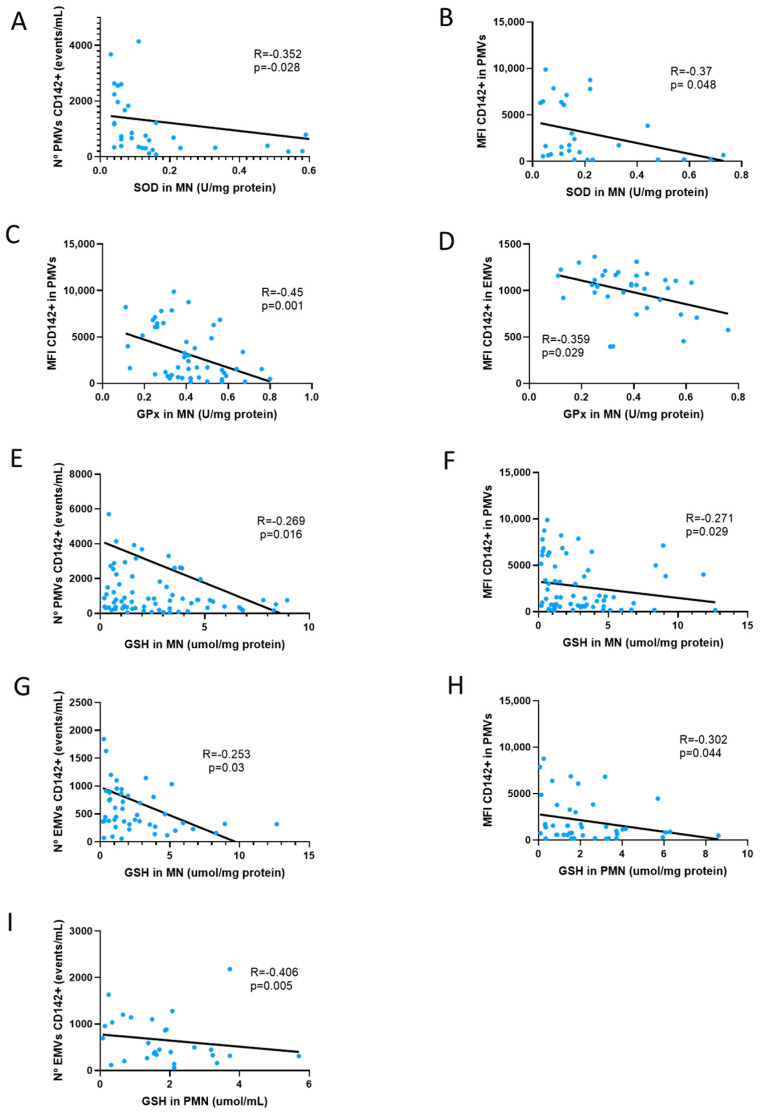
Antioxidant activity and TF expression in MVs. Correlation between SOD in MN and the total number of CD142+PMVs (**A**) and MFI of CD142 expression on PMVs (**B**). Correlation between GPx in MN and MFI of CD142 expression in PMVs (**C**) and EMVs (**D**). Correlation between GSH in MN and the total number of CD142+PMVs (**E**), MFI of CD142 expression on PMVs (**F**) and EMVs (**G**). Correlation between GSH in PMN and MFI of CD142 expression on PMVs (**H**) and EMVs (**I**) (Spearman correlation). SOD, superoxide dismutase; GPx, glutathione peroxidase; GSH, reduced glutathione; MN, mononuclear leukocytes; PMN, polymorphonuclear leukocytes; PMVs, platelet microvesicles; MFI, mean fluorescence intensity; EMVs, endothelial microvesicles. The total number of ACKD, HD and PD patients with or without APA therapy were analyzed. Sample size: 40 ACKD, 40 HD, 36 PD and 17 HS).

The correlations between the antioxidant parameters and TF expression in EMVs and PMVs are shown in Figure 7. ACKD, HD and PD patients with or without APA therapy were analyzed. The number of PMVs expressing TF and the expression (MFI) of TF in PMVs were negatively correlated with SOD activity in MN (r = −0.352, *p* = 0.028 and r = −0.37, *p* = 0.048; Figure 7A,B). The expression (MFI) of TF in PMVs and the number of EMVs expressing TF showed a negative correlation with GPx activity in MN (r = −0.045, *p* = 0.001 and r = −0.359, *p* = 0.029; Figure 7C,D). The number of PMVs expressing TF, the expression (MFI) of TF on PMVs, and the number of EMVs expressing TF were negatively correlated with the amount of GSH in the MN (r = −0.269, *p* = 0.016; r = −0.271, *p* = 0.029 and r = −0.253, *p* = 0.03; Figure 7E–G). The expression (MFI) of TF in PMVs and the number of EMVs expressing TF showed a negative correlation with the amount of GSH in PMN (r = −0.302, *p* = 0.044 and r = −0.406, *p* = 0.005; Figure 7H,I). The remaining correlations between the antioxidant parameters and TF expression in EMVs and PMVs are shown in Table A4 and Table A5.

**Figure 8:**
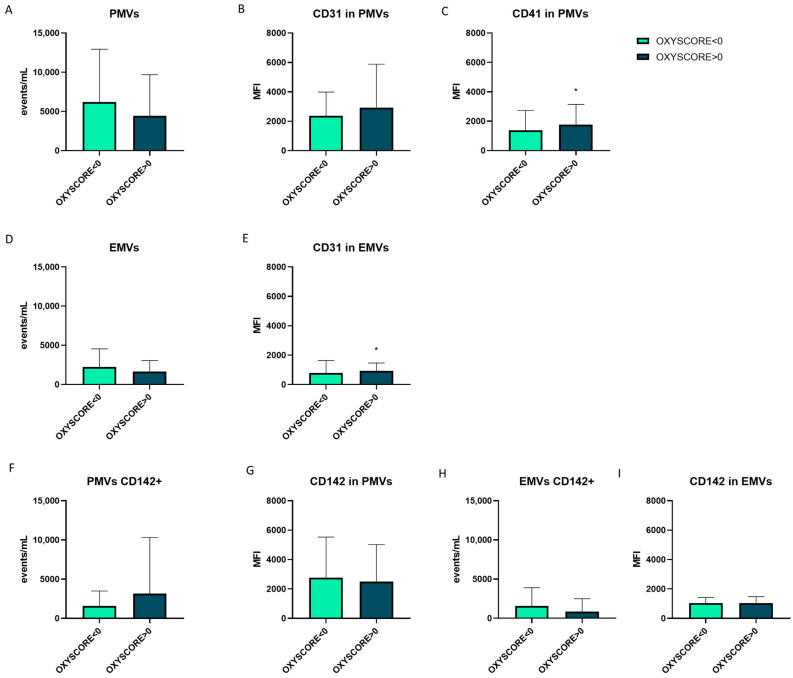
OXY-SCORE levels are related to the expression of CD41 and CD31 in PMVs and EMVs. Comparison between PMVs and EMVs phenotypes and OXY-SCORE levels. Comparison between OXY-SCORE levels and number of PMVs (**A**), expression of CD31 in PMVs (**B**), expression of CD41 in PMVs (**C**), number of EMVs (**D**), expression of CD31 in EMVs (**E**), number of PMVs expressing CD142/TF (**F**), expression of CD142/TF in PMVs CD142+ (**G**), number of EMVs expressing CD142/TF (**H**) and expression of CD142/TF in EMVs CD142+ (**I**). PMVs: platelet microvesicles; EMVs: endothelial microvesicles: MFI: mean fluorescence intensity. * *p* < 0.05 vs. OXY-SCORE < 0. Statistical analysis: Student’s *t*-test and Mann–Whitney U test were used. The total number of ACKD, HD and PD patients with or without APA therapy were analyzed.

The release and phenotypes of the PMVs and EMVs were analyzed in relation to an individualized OXY-SCORE. ACKD, HD and PD patients with or without APA therapy were analyzed. In patients with CKD and an OXY-SCORE score above 0, higher CD41 expression was observed in PMVs (Figure 8C) and higher CD31 expression in EMVs (Figure 8E). No differences were observed in the number of PMVs (Figure 8A), expression of CD31 in PMVs (Figure 8B), number of EMVs (Figure 8D), number of PMVs expressing TF (Figure 8F), expression of TF in PMVs CD142+ (Figure 8G), number of EMVs expressing TF (Figure 8H) and expression of TF in EMVs CD142+ (Figure 8I).

**Figure 9:**
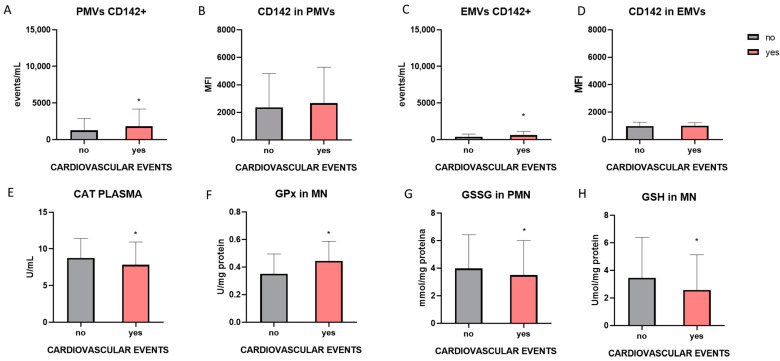
Increased TF expression in MVs is related to cardiovascular events (“yes” in the graphical representation and “no” is associated with no cardiovascular events). Related to cardiovascular events total number of CD142+PMV (**A**), CD142 expression on PMV (**B**), total number of CD142+EMV (**C**) and CD142 expression on EMVs (**D**). The levels of catalase in plasma (**E**), GPx in MN (**F**), GSSG in PMN (**G**), and GSH (**H**) were related to cardiovascular events. * *p* < 0.05 vs. no; (Statistical analysis: Student’s *t*-test and Mann–Whitney U test were used). PMVs, platelet microvesicles; EMVs, endothelial microvesicles; CAT, catalase; GPx, glutathione peroxidase; GSSG, glutathione disulfide; GSH, reduced glutathione; MFI, mean fluorescence intensity. The total number of ACKD, HD and PD patients with or without APA therapy were analyzed.

The possible relationship between the parameters studied and the occurrence of cardiovascular events was analyzed. ACKD, HD and PD patients with or without APA therapy were analyzed. Subjects who had experienced cardiovascular events before or during the following 5 years of follow-up showed elevated levels of PMVs CD142+ (Figure 9A) and EMVs CD142+ (Figure 9C), with a trend in CD142 expression between PMVs (MFI) (Figure 9B) and with no differences in the expression of CD142+ in EMVs (MFI) (Figure 9D). Subjects who had suffered cardiovascular events prior to or during the 5-year follow-up showed elevated levels of GPx in the MN (Figure 9F) and GSSG in the PMN (Figure 9G), as well as lower levels of CAT and GSH (Figure 9E,H).

## 4. Discussion

CKD is associated with a high incidence of CVD due to the accumulation of uremic toxins, an altered REDOX state, and chronic systemic inflammation [19]. These factors, together with underlying endothelial damage, significantly increase the risk of acute events and pathologies of cardiovascular and thrombotic origins. However, there are no known reliable biomarkers that can demonstrate the underlying damage and predict and prognosticate cardiovascular events in CKD [20]. Among the factors scarcely in the context of CKD are MVs of endothelial and platelet origin, as well as their relationship with different parameters related to the patient’s REDOX status. Our study aimed to establish for the first time the existence of this relationship, as well as to analyze the individual REDOX status and study in depth the role of both factors in the development of CVD in renal patients.

Previous studies by our group have reported alterations in oxidative stress parameters in CKD [16]. To assess the oxidative stress of the study subjects as accurately as possible and on an individual basis, we applied the OXY-SCORE developed by [17] to the data obtained. When studying the effect of the different variables together on patients, we obtained a higher OXY-SCORE in ACKD and HD, as well as an OXY-SCORE in PD similar to HS. Overall, patients with PD appear to have lower oxidative stress or an increased ability to reach a redox balance. In patients with ACKD and HD, OXY-SCORE is higher, which indicates greater oxidative stress, since the pro-oxidant parameters are elevated and the antioxidants are reduced. The results in PD may indicate that in patients with high levels of pro-oxidant parameters there are high levels of antioxidants that counteract them, inducing an oxidative state similar to HS.

To explain the OXY-SCORE found in the different groups, we return to the results of the cross-sectional study [16], the development of which has been continued, increasing the sample size and whose results are shown in Table A1 and Table A2, where we can see elevated levels of pro-oxidant parameters (XO, TBARS, GSSG and GSSG/GSH ratio) in CKD in plasma as well as in MN and PMN. These elevated levels may correspond to nutritional restrictions, malnutrition, as well as accumulated uremic toxins not eliminated by dialysis, which lead to the activation of leukocytes, producing more pro-oxidant enzymes [20,21]. Circulating XO induces phagocytosis and the production of O_2_^−^ and H_2_O_2_, which can be distributed to distant tissues or enter cells causing oxidative damage [22]. Furthermore, in our study, we observed that plasma CAT levels did not increase, which could indicate that the accumulation of H_2_O_2_ in CKD is not resolved, inducing lipid peroxidation together with other ROS. TBARS levels were elevated in the plasma in response to an increase in ROS such as H_2_O_2_ [16]. TBARS levels are elevated in heart failure and have been associated with a worse prognosis, which could indicate poor evolution of CVD in patients with CKD [23]. The GSSG per mg of protein, as well as the GSH per mg of protein in MN and PMN, provide information on the degree of protein oxidation. Our results showed elevated levels of GSSG in all the study groups, indicating a high degree of protein oxidation. GSH levels were particularly elevated in HD and PD patients, which could be due to a protective factor. These alterations are due to the accumulation of uremic toxins and the deficient clearance of some of them by dialysis techniques [24]. GSH prevents the accumulation of ROS such as O_2_^−^ and H_2_O_2_, and GSSG is a toxic product in cells, both of which are considered important indicators of damage and maintenance of immune functions [25].

In general, we also observed a decrease in antioxidant parameters (GPx, CAT, GSH and SOD) in CKD. SOD levels decreased in MN leukocytes. This enzyme catalyzes the transformation of O_2_^−^ into H_2_O_2_. As mentioned, H_2_O_2_ induces lipid peroxidation; therefore, lower SOD levels could explain why MDA levels were similar to HS in MN and PMN in all study groups. CAT and GPx catalyze the transformation of the toxic product H_2_O_2_ into H_2_O. GPx also catalyzes the reduction of lipid products and protects against lipid peroxidation [26]. This could explain why, in plasma, where we observed reduced levels of GPx (as a trend and evidence in PD) we observe elevated levels of TBARS, whereas in leukocytes, where we did not observe differences in GPx levels, we also did not see differences in the amount of MDA.

Biomolecules such as citrate and acetate in dialysis system fluids are also relevant in CKD. It has been described that acetate favors greater leukocyte activation, triggering a greater release of ROS, while citrate increases antioxidant enzymes such as SOD and reduces the levels of pro-oxidant and lipid peroxidation components, evidenced by a decrease in levels of lipid peroxidation products such as MDA [27]. The components of PD fluids, as well as their exposure to the peritoneal membrane have also been shown to promote oxidative stress. Increasing glucose or lactate concentrations and hyperosmolarity of the dialysate promotes the production of ROS, which accumulate in the peritoneal membrane, causing calcification and fibrosis of the peritoneal membrane [28].

In order to investigate the causes of the differences in individualized OXY-SCORE values between ACKD and different CKD treatments, the mechanisms that inhibit antioxidant enzyme synthesis in ACKD and HD, which do not seem to occur in PD, need to be explored further. The OXY-SCORE developed by [17] seems to be applicable to the parameters studied in the present study. An optimal oxidative state is essential for the maintenance of the correct functioning of the organism, especially the immune system [29].

In contrast, in patients with CKD, the endothelium is damaged, modifying the vascular structure and the expression of adhesion molecules, which favors platelet adhesion, initiating the coagulation process, and favoring inflammation and thrombotic processes. Endothelial damage may constitute the initial trigger for cardiovascular events, and there are numerous factors, such as oxidative stress and the release of MVs, which may play a role in its development [30]. In patients with CKD, in whom CVD is the main cause of mortality, there may be underlying endothelial damage, which is difficult to detect and evaluate in clinical practice, and platelet hyperactivation, which increases the risk of thrombosis. Our group has proposed the study of EMVs and PMVs, as well as their expression of membrane molecules, as a method to evaluate these issues. In our study, a greater number of plasma PMVs and a greater expression of CD41 were observed in patients with PD. HD subjects showed a greater number of EMVs in plasma, as well as a greater expression of CD31. Finally, our results showed a higher number of PMVs and EMVs expressing TF as well as a higher expression of TF by MVs in patients with ACKD.

In CKD, due to the accumulation of uremic toxins and other factors inherent to the disease, such as inflammation, there is an increase in platelet activation and the release of PMVs, showing increased procoagulant activity [31]. In our study, PD patients showed elevated levels of PMVs, but this was not the case for ACKD and HD. Previous studies have shown elevated levels of PMVs in HD [32], which may be due to the use of drugs or factors corresponding to HD or particularities in relation to HD techniques that respond to the protocols of each hospital, such as a more widespread use of online HD or differences in dialysis fluids. In our study, we did not establish differences in terms of the use of different drugs apart from APA, and we must proceed to study different combinations of drugs and other factors inherent to HD, as well as different hemodialysis techniques or the use of different materials in the membranes. Although in our study there was no increase in plasma PMVs in ACKD, they did express more CD41 and TF, unlike the rest of the groups, where the expression was similar to HS, or, in the case of TF in HD, even lower than in HS. This may be because although the drugs used in ACKD seem to act on the release of PMVs, they do not seem to affect the expression of TF and CD41, which is higher in ACKD, causing an increased thrombotic risk. Factors that reduce the release of PMVs in HD also appear to reduce CD41 and TF expression. This may be related to the accumulation of uremic toxins in ACKD, which are cleared by dialysis [33]. Considering that both the release of PMVs and their expression of CD41 and TF may indicate greater platelet activity, these results indicate a higher prothrombotic risk in ACKD and PD than in HS [34,35]. PMVs are involved in coagulation, tissue repair, inflammation, immune response and healing [14].

In this study, the number of EMVs was higher in the HD group than that in the other groups. Other authors have postulated that in ACKD, the combination of statins and APA reduces the release of EMVs, with elevated levels observed in HD despite the clearance of uremic toxins [36]. This may be due to numerous factors, such as the persistence of reduced renal function, use of non-biocompatible materials, presence of vascular accesses, or hemodynamic stress, with a wide dispersion [37]. In addition to showing a high number of EMVs in HD, they also express a higher amount of CD31. CD31, an endothelial adhesion molecule, is expressed in greater quantities close to the junctions between endothelial cells, which may not only represent a greater adhesion capacity, but also evidence of a greater loss of endothelial structure in HD [37]. In ACKD, we also observed, as was already the case in PMVs, that a greater number of EMVs expressed TF, but also a greater expression of TF per MVs. This could indicate an increased thrombotic risk in patients with ACKD. However, the effect of HD on MV release remains controversial. Several authors argue for an increase in circulating MVs in HD [37], because of the biocompatibility of the components involved, whereas other studies show no significant difference or even a decrease in the number of MVs, attributed to the adhesion of MVs to dialysis filters [38]. This question needs to be the subject of further studies to verify the role of TF in the development of CVD. Prospective studies might be carried out in order to analyze TF’s role as a biomarker of prognosis and diagnosis of CVD in CKD.

We established a relationship between the release of MVs and their phenotype with oxidative stress parameters, as other authors have proposed a relationship in other cells, such as erythrocytes and uterine cells [39,40]. We observed a positive correlation between XO activity in MN and PMN and the expression of TF in EMVs and PMVs, as well as a negative correlation between SOD activity, GPx activity, the amount of GSSG per mg protein and GSH per mg of protein in MN and PMN and the expression of TF in EMVs and PMVs.

Although the relationship between EMVs and PMVs and the patient REDOX state has not been previously studied, it has been observed that MVs may participate in the production or detoxification of ROS [40]. Additionally, ROS may be involved in MV production. The molecular mechanisms activated by MVs are not fully understood, although the involvement of miRNAs, which are small RNAs contained in MVs that regulate the gene expression of target cells, has been defended [41]. It has been shown that some miRNAs, such as miRNA-126 and miRNA-21, are involved in oxidative stress and activating or inhibiting signaling pathways, the study of which is currently booming [42,43]. Other authors have proposed the possibility of the coexistence of both types of pathways, as they have demonstrated through the use of cell cultures that endothelial cells produce MVs that remove ROS but also MVs that induce the production of ROS as part of the physiological signaling processes of endothelial cells [44].

Our study showed a negative correlation between the amount of GSSG per milligram of protein and the expression of TF in EMVs and PMVs. This may be because a higher amount of GSSG reflects the higher activity of GPx in transforming H_2_O_2_ into H_2_O. During this process, GSH is transformed into GSSG; therefore, a higher amount of GSSG per mg protein would be evidence of a higher GPx antioxidant capacity. This greater antioxidant capacity would have an impact on TF expression, thereby reducing it [45].

OXY-SCORE showed a relationship with the expression of CD41 in PMVs and the expression of CD31 in EMVs, which may indicate that this expression is conditioned by the REDOX state, whereas the expression of TF is not as conditioned by the REDOX state as it is by specific parameters involved in oxidative imbalance, such as XO, GPx, SOD, GSSG, and GSH. Although the relationship between CD41 expression in PMVs and CD31 expression in EMVs and OXY-SCORE, which establishes the individualized REDOX status of patients, has not been analyzed in previous studies, a relationship has been reported with different parameters that show the degree of oxidative stress, such as the amount of ROS, isolated parameters such as SOD activity, the relationship between CD41 expression in platelets, and the degree of oxidation of lipoproteins [46]. Our results suggest a different effect of the general REDOX state of a patient, as evidenced by OXY-SCORE, and specific pro-oxidant and antioxidant parameters on the expression of CD41, CD31 and TF in PMVs and EMVs, which may be due to different pathways activated or inhibited by an altered REDOX state or by the action of specific enzymes or elements such as the products of lipid peroxidation. To answer this question further, we propose in vitro studies that analyze in depth the pathways that may be involved.

Patients with CKD often require APAs to prevent thrombotic pathologies associated with the disease. APAs inhibit platelet activation and aggregation via different pathways, thereby preventing the formation of thrombi. In CKD, one of the most commonly used APAs is acetylsalicylic acid (ADIRO), which was used in the treatment of patients in our study. Acetylsalicylic acid works by inhibiting the synthesis of thromboxane A2 (platelet aggregation agonist) [47]. Regarding the role of APA drugs, in our study it was observed that APA had a beneficial effect in reducing TF expression in PEVs and EMVs in PD. However, previous studies on this topic have been limited. As in our study, no effect on the release of PMVs and EMVs was observed, although a decrease in smooth muscle-derived MVs expressing TF has been observed in previous studies [47]. Our data showed a decrease in PMVs expressing TF in PD, which may mean that the use of APA might decrease the risk of thrombotic events in patients with CKD. The causes of the decrease in TF expression in PD and not in the other groups are unknown; however, our group postulates the activation of different pathways that affect TF expression with differential modulation by APA, a line that could surely benefit from future studies.

APAs do not appear to affect the production of pro-oxidant and antioxidant parameters in MN or PMN, although the behavior in plasma is confusing and appears to have a reducing effect on GPx in ACKD and TBARS in HD, as well as being related to higher levels of XO in PD (Table A1 and Table A2). Since there have been no previous studies relating the use of these drugs to the above-mentioned parameters, our group proposed that the observed differences could be due to side effects of APA on platelets and proposed in vitro studies to investigate this question further.

Finally, we sought to relate the analyzed parameters to the occurrence of cardiovascular events. Patients who had experienced cardiovascular events before sampling or during the 5 years of follow-up had elevated levels of TF expression compared to those who had never experienced cardiovascular events. The increased expression of TF has been linked to different pathologies or events of cardiovascular origin, due to its role in coagulation. Cardiovascular events are associated with lower levels of antioxidant factors such as CAT and GSH, as well as elevated levels of GPx, which may be a protective mechanism, and elevated levels of GSSG. Oxidative stress has been previously related to the occurrence of cardiovascular events due to the relationship between different parameters of oxidative stress and ROS with inflammatory or atherosclerosis-related parameters [48].

One of the main objectives of our research is to identify and validate non-invasive biomarkers of the pathological processes underlying cardiovascular damage in clinical practice for kidney patients. From our biomarker profile it will be possible to develop a more accurate clinical profile of each patient, which will allow personalized treatment. In addition, the use of the different techniques and methodologies employed, such as biochemical determinations or flow cytometry for phenotyping of VMs, are routinely available and implemented in the hospital setting for other determinations, which does not entail an increase in economic costs or specialized personnel for their performance. The validation of personalized biomarker profiles is safe in the diagnosis and progression of the disease and, in the long run, is less costly for the healthcare system.

To ensure that our methodology in the phenotypic characterization of MVs by flow cytometry complied with the standards of sensitivity, accuracy, robustness, stability, specificity and linearity of the results, we have counted on the experienced and qualified support of the CCMF of the UCM. CCMF is a reference center, with internal and external quality controls that guarantee the validity of the technique for the objectives of this study. In addition, the specialized staff has guided us in all the procedures and supervised the results obtained. Due to the high cost of care in the progression of renal disease and the high incidence of CVD associated with CKD, the search for biomarkers, easily and quickly determined for prognosis, prediction and early diagnosis, would add an additional economic benefit to the health system in these patients.

This study has some limitations. Increasing the sample size would allow us to deepen our understanding of the relationship between MVs and oxidative stress parameters. It would also allow us to delve into the analysis of the different types of APAs, as well as the effect of anticoagulants and other drugs that may have pleiotropic effects. A multicenter study including more than one hospital could be interesting to carry out.

## 5. Conclusions

The data obtained in this study concluded that, in subjects with CKD (ACKD, HD, and PD), there were alterations in the REDOX state. However, the use of an individualized OXY-SCORE represents a more reliable measure for accurately assessing the REDOX status of patients. In CKD, there are alterations in the functionality of the endothelial cells and platelets. These alterations can be evidenced by the analysis of PMVs and EMVs, as well as the expression of surface molecules, such as CD31, CD41 and TF, which provide information about the activity of the emitting cells, as well as their possible action on target cells. Based on these parameters, the present study showed an increased prothrombotic risk in patients with ACKD and PD as well as increased underlying endothelial damage in HD. The expression of TF appears to be related to some parameters of oxidative stress, although more extensive studies are needed to determine whether this is a feedback relationship. However, the oxidative stress evidenced by individualized OXY-SCORE seems to be related to the expression of CD31 and CD41 in PMVs and EMVs, respectively. Finally, some effects of APAs on TF expression seems to be demonstrated, as patients who have suffered cardiovascular events have low levels of antioxidant parameters and higher TF expression. Therefore, we postulate that MVs and oxidative stress parameters can be used as markers of cardiovascular and thrombotic risk. We propose the expression of TF in PMVs and EMVs as possible predictive and prognostic biomarkers of CVD and acute cardiovascular events, as well as the role of APAs as a possible beneficial factor for reducing this risk.

## Figures and Tables

**Table 1:** Demographic and clinical characteristics of the study population.

Characteristics	HS (*n* = 15)	ACKD (*n* = 40)	HD (*n* = 40)	PD (*n* = 36)
Demographic data				
Age (years; mean ± SE)	51 ± 15.5	60.7 ± 17.2	57.4 ± 14.6	56.2 ± 13.4
Nº women, *n* (%)	8 (47)	14 (35)	13 (32.5)	17 (47.2)
Smoking, *n* (%)	3 (18)	11 (27.5)	16 (40)	9 (25)
eGFR (mL/min/1.73 m^2^)	69 ± 10.53	14.04 ± 4.9 *	7.36 ± 4.08 ***^###^	7.14 ± 2.63 ***^###^
KT/V	-	-	1.67 ± 0.25	2.34 ± 0.5
Comorbidity				
Diabetes mellitus, *n* (%)	0 (0)	18 (45) **	7 (15.5)	11 (30.6) *
Dyslipidemia, *n* (%)	0 (0)	31 (77.5) ***	23 (57.5) ***	22 (61.1) ***
Hyperuricemia, *n* (%)	0 (0)	28 (70) ***	11 (27.5) **	19 (52.8) ***
Hypertension, *n* (%)	1 (6)	36 (90) ***	33 (82.5) ***	33 (91.7) ***
Cardiovascular events, *n* (%)	0 (0)	22 (55) **	22 (55) **	20 (55.6) **
*Ischemic cardiopathy (n (%))*	0 (0)	17 (42.5) **	21 (52.5) ***	16 (44.4) ***
*Acute cardiovascular accident (n (%))*	0 (0)	6 (15) *	2 (5)	8 (22.2) *
*Vasculopathy (n (%))*	0 (0)	4 (10)	15 (37.5) **	5 (13.8) *
*Chronic cardiac insufficiency (n (%))*	0 (0)	3 (7.5)	7 (17.5) *	4 (11.1)
Treatment				
Erythropoietin, *n* (%)	0 (0)	19 (47.5) ***	40 (100) ***	20 (55.6) ***
Statin, *n* (%)	0 (0)	30 (75) ***	16 (40) **	19 (52.8) ***
Alopurinol, *n* (%)	0 (0)	24 (60) ***	8 (20) **	17 (47.2) ***
Antiplatelets agents, *n* (%)	0 (0)	7 (17.5) *	10 (25) *	7 (19.4) *
Anticoagulants, *n* (%)	0 (0)	8 (20) *	5 (12.5)	1 (2)

HS, healthy subjects; ACKD, advanced chronic kidney disease; HD, hemodialysis; PD, peritoneal dialysis; eGFR, glomerular filtration rate; KT/V, amount of urea removed in one session/volume distribution of urea in a patient; SD, standard deviation. * *p* < 0.05; ** *p* < 0.01; *** *p* < 0.01; ^###^ *p* < 0.001 vs. ACKD. Chi-square test.

## Data Availability

Data is contained within the article.

## Appendix A

**Table A1:** Pro-oxidant and antioxidant parameters in plasma.

Plasma							
	HS	ACKD (Non APA)	ACKD (APA)	HD (Non APA)	HD (APA)	PD (Non APA)	PD (APA)
XO activity in plasma (mU/mL)	0.04 ± 0.01	0.05 ± 0.3	0.036 ± 0.005	0.05 ± 0.039	0.057 ± 0.03	0.06 ± 0.02	**0.09 ± 0.07 ^a^**
TBARS (nmol/mL)	5.6 ± 2.8	11.4 ± 7.7	11.2 ± 6.18	**12.25 ± 6.39 ****	**8.8 ± 3.7 ^##^**	**14.4 ± 8.9 ****	**16.1 ± 9.8 **^##^**
GPx activity (U/mL)	60.2 ± 15.6	32.9 ± 9.7	29.9 ± 12.7	35.2 ± 18.3	32.3 ± 13.1	44.6 ± 6.5	**33.3 ± 8.3** *^##^
GSH (umol/mL)	1.8 ± 0.53	1.26 ± 0.62	1.17 ± 0.53	**0.9 ± 0.34 ****	1 ± 0.3	**0.96 ± 0.34 ****	**1 ± 0.56 ***
CAT activity in plasma (U/mL)	11.4 ± 4.9	10.2 ± 2.6	**6.47 ± 2.85 ^a^**	9.6 ± 3.12	9.2 ± 2.8	7.3 ± 2.4	6 ± 2.5
SOD (U/mL)	0.65 ± 1.47	1.3 ± 1.5	0.38 ± 0.27	0.82 ± 0.59	0.69 ± 0.61	**1.4 ± 1.5 ^$^**	0.79 ± 0.98

XO, xanthine oxidase; GPx, glutathione peroxidase; TBARS, Thiobarbituric acid reactive substances; GSH, Glutathione; CAT, catalase; SOD, superoxide dismutase; HS, healthy subjects; ACKD, advanced chronic kidney disease; HD, hemodialysis; PD, peritoneal dialysis; APA, antiplatelets agents. * *p* < 0.05 vs. HS; ** *p* < 0.01 vs. HS; ^##^ *p* < 0.01 vs. ACKD; ^$^ *p* < 0.05 vs. HD; ^a^ *p* < 0.05 vs. non-APA.

**Table A2:** Pro-oxidant and antioxidant parameters in mononuclear and polymorphonuclear leukocytes.

Mononuclear Leucocytes								Polimorphonuclear Leucocytes						
	HS	ACKD (Non APA)	ACKD (APA)	HD (Non APA)	HD (APA)	PD (Non APA)	PD (APA)	HS	ACKD (Non APA)	ACKD (APA)	HD (Non APA)	HD (APA)	PD (Non APA)	PD (APA)
XO activity (mU/mg proteins)	2.7 ± 1.08	3.7 ± 1	3.12 ± 0.88	**1.55 ± 0.89 *^###^**	**1.7 ± 1.3 *^###^**	3.7 ± 1.2	**5.1 ± 2.9 *^$$$^**	2.4 ± 1.8	**3.8 ± 2.25 ***	**4.59 ± 2.5 ****	**1.1 ± 1.1 *^###^**	**1 ± 0.76 *^###^**	**2.7 ± 1.2 ^$$$^**	**2.3 ± 0.46 ^$$^**
MDA (nmol/mg proteins)	2.2 ± 0.9	3.11 ± 2.82	4.1 ± 2.3	3.59 ± 1.65	2.6 ± 1.1	2.8 ± 1.7	2.7 ± 1.6	3.3 ± 2	3.41 ± 2.12	2.2 ± 1.1	2.82 ± 1.57	2.6 ± 1.3	2.4 ± 1.5	2.3 ± 0.85
GPx activity (U/mg proteins)	0.37 ± 0.08	0.38 ± 0.19	0.44 ± 0.15	0.42 ± 0.13	0.56 ± 1.14	0.36 ± 0.09	0.35 ± 0.1	0.33 ± 0.11	0.45 ± 0.18	0.42 ± 0.11	0.32 ± 0.1	0.36 ± 0.077	0.13 ± 0.14	**0.64 ± 0.12 ***
CAT activity (U/mg proteins)	9.4 ± 2.5	7.9 ± 2	-	**5.84 ± 1.44 ****	**5.9 ± 1.3 ****	**5.9 ± 1.25 ****	7 ± 1.7	7.8 ± 1.7	**5 ± 1.6 *****	**5.17 ± 2.61 *****	**3.96 ± 0.84 *****	**4.6 ± 1.6 *****	6.3 ± 1	**5.9 ± 0.09 ****
SOD (U/mg proteins)	0.55 ± 0.3	**0.11 ± 0.06 *****	**0.09 ± 0.06 *****	**0.37 ± 0.26 ^#^**	**0.3 ± 0.2 ^#^**	**0.15 ±** **0.18 ***^$$^**	**0.08 ± 0.04 ***^$$^**	0.23 ± 0.2	0.35 ± 0.54	0.35 ± 0.34	0.49 ± 0.37	0.4 ± 0.5	0.15 ± 0.15	0.3 ± 0.3
GSSG (nmol/mg proteins)	3.2 ± 2.2	2.66 ± 2.47	**3.4 ± 2** ^a^	**4 ± 2 ^#^**	**4.1 ± 2.6 ^#^**	**5.9 ± 2.4 ^##^**	3.7 ± 1.2	0.2 ± 0.07	**1.62 ± 1.11 *****	**3.3 ± 0.9 ***^a^**	**1.22 ± 0.51 *****	**1.8 ± 2.4 *****	**0.86 ± 0.34 ****	**0.94 ± 0.54 ****
GSH (umol/mg proteins)	5.9 ± 5.2	**2.62 ± 2.47 *****	**2.1 ± 1.4 *****	4 ± 3	**3.5 ± 3 ^#^**	3 ± 3.3	2.9 ± 1.66	1.3 ± 2.4	1.05 ± 0.95	1.31 ± 0.46	**3.8 ± 2.3 ***^###^**	**3.8 ± 2.5 ***^###^**	**2.7 ± 2.18 **^##^**	**3.3 ± 1.09 **^##^**
GSSG/GSH	0.9 ± 0.98	1.17 ± 0.88	1.7 ± 1	1.69 ± 1.55	**2.7 ± 3 ***	**3.4 ± 2.11 ****	**3.2 ± 6.4 ****	0.36 ± 0.26	**4.32 ± 5.75 *****	2.7 ± 1.2	**0.56 ± 0.58 ^###^**	**0.83 ± 1.29 ^###^**	**0.8 ± 0.99 ^###^**	**1.1 ± 2 ^##a^**

XO, xanthine oxidase; GPx, glutathione peroxidase; MDA, malondialdehyde; CAT, catalase; SOD, superoxide dismutase; GSSG: Glutathione disulfide; GSH; Glutathione; HS (CT), healthy subjects; ACKD, advanced chronic kidney disease; HD, hemodialysis; PD, peritoneal dialysis; APA, antiplatelets agents. * *p* < 0.05 vs. HS; ** *p* < 0.01 vs. HS; *** *p* < 0.001 vs. HS; ^#^ *p* < 0.05 vs. ACKD; ^##^ *p* < 0.01 vs. ACKD; ^###^ *p* < 0.05 vs. ACKD; ^$$^ *p* < 0.01 vs. HD; ^$$$^ *p* < 0.001 vs. HD; ^a^ *p* < 0.05 vs. non-APA.

**Table A3:** Microvesicles levels and phenotypes.

	HS	ACKD (NON APA)	ACKD (APA)	HD (NON APA)	HD (APA)	PD (NON APA)	PD (APA)
N° PMVs (events/mL)	4468 ± 2485	2878 ± 1664	3995 ± 4229	3052 ± 2034	4112 ± 4124	5447 ± 4684	**8970 ± 5653 ^##$$^**
CD31 in PMVs (MFI)	1910 ± 679	3070 ± 1966	3504 ±2165	2168 ± 1476	2632 ± 1425	1498 ±581	2156 ± 2538
CD41 in PMVs (MFI)	987 ± 386	**2436 ± 1335 *****	2754 ± 2048	**997 ± 612 ^###^**	1640 ± 1366	**785 ± 514 ^###^**	**551 ± 278 ^#^**
N° EMVs (events/mL)	1371 ± 675	796 ± 471	597 ±412	**2272 ± 1242 ^#^**	**1918 ± 1438 ^##^**	**1739 ± 1185 ^$^**	1487 ± 1200
CD31 in EMVs (MFI)	951 ± 327	718 ± 240	721 ± 241	842 ± 182	1136 ± 501	**525 ±298 ***^$^**	**564 ± 344 ^$$^**
N° PMVs CD142 + (events/mL)	878 ± 409	1948 ± 1340	2915 ± 701	**376 ± 215 ^###^**	**909 ± 866 ^##^**	**1216 ± 850 ^$$^**	**798 ± 576 ^##a^**
CD142 in PMV CD142 + (MFI)	1878 ± 814	**3990 ± 3121** *	2335 ± 1991	**1379 ± 523 ^##^**	1738 ± 1932	1643 ± 970	1269 ± 677
N° EMVs CD142 + (events/mL)	210 ±157	**652 ± 457 ***	713 ± 309	**279 ± 266 ^#^**	370 ± 262	329 ± 203	165 ± 55
CD142 in EMV CD142 + (MFI)	744 ± 223	**1185 ± 594 ****	1000 ± 207	**1167 ± 87 ****	1001 ± 166	935 ± 492	848 ± 296

HS, healthy subjects; ACKD, advanced chronic kidney disease; HD, hemodialysis; PD, peritoneal dialysis; PMVs, platelet microvesicles; EMVs, endothelial microvesicles; APA, antiplatelets agents. * *p* < 0.05 vs. HS; ** *p* < 0.01 vs. HS; *** *p* < 0.001; ^#^ *p* < 0.05 vs. ACKD non APA; ^##^ *p* < 0.01 ACKD non APA; ^###^ *p* < 0.05; ^$^ *p* < 0.05 vs. HD non APA; ^$$^ *p* < 0.01 vs. HD non APA; ^a^ *p* < 0.05 vs. PD non APA.

**Table A4:** Correlation between pro-oxidant and antioxidant variables and MVs release in the plasma.

	XO Activity in Plasma (mU/mL)	TBARS (nmol/mL)	GPx Activity (U/mL)	GSH (umol/mL)	CAT Activity in Plasma (U/mL)	SOD (U/mL)
N° PMV	0.115 (*p* = 0.309)	0.09 (*p* = 0.41)	−0.191 (*p* = 0.131)	−0.039 (*p* = 0.735)	0.14 (*p* = 0.298)	0.149 (*p* = 0.208)
CD31 IN PMV	−0.197 (*p* = 0.102)	0.059 (*p* = 0.614)	−0.021 (*p* = 0.882)	0.207 (*p* = 0.095)	0.084 (*p* = 0.569)	0.097 (*p* = 0.451)
CD41 IN PMV	−0.058 (*p* = 0.621)	0.114 (*p* = 0.31)	−0.253 (*p* = 0.051)	**0.357 **** **(*p* = 0.002)**	0.072 (*p* = 0.603)	0.055 (*p* = 0.653)
N° EMV	0.045 (*p* = 0.704)	0.068 (*p* = 0.55)	−0.162 (*p* = 0.226)	−0.119 (*p* = 0.319)	**0.293 *** **(*p* = 0.037)**	0.153 (*p* = 0.215)
CD31 IN EMV	−0.186 (*p* = 0.106)	−0.03 (*p* = 0.786)	−0.091 (*p* = 0.487)	0.077 (*p* = 0.521)	0.07 (*p* = 0.617)	−0.161 (*p* = 0.186)
N° PMV CD142+	0.087 (*p* = 0.432)	−0.103 (*p* = 0.335)	−0.044 (*p* = 0.723)	0.120 (*p* = 0.283)	−0.087 (*p* = 0.503)	0.004 (*p* = 0.97)
CD142 IN PMV	0.027 (*p* = 0.829)	−0.017 (*p* = 0.887)	0.009 (*p* = 0.949)	**0.314 *** **(*p* = 0.01)**	−0.089 (*p* = 0.558)	0.054 (*p* = 0.677)
EMV CD142+	0.168 (*p* = 0.144)	−0.146 (*p* 0.19)	−0.03 (*p* = 0.822)	0.201 (*p* = 0.091)	0.022 (*p* = 0.872)	0.087 (*p* = 0.476)
CD142 IN EMV	−0.024 (*p* = 0.868)	0.016 (*p* = 0.907)	−0.094 (*p* = 0.584)	0.228 (*p* = 0.107	0.175 (*p* = 0.322)	0205 (*p* = 0.172)

XO, xanthine oxidase; GPx, glutathione peroxidase; MDA, malondialdehyde; CAT, catalase; SOD, superoxide dismutase; GSH; Glutathione; PMVs, platelet microvesicles; EMVs, endothelial microvesicles. * *p* < 0.05; ** *p* < 0.01.

**Table A5:** Correlation between pro-oxidant and antioxidant variables and MVs release in leucocytes.

	XO IN MN (mU/mg Proteins)	XO IN PMN (mU/mg Proteins)	CAT IN MN (U/mg Proteins)	CAT IN PMN (U/mg Proteins)	SOD IN MN (U/mg Proteins)	SOD IN PMN (U/mg Proteins)	GPx IN MN (U/mg Proteins)	GPx IN PMN (U/mg Proteins)	MDA IN MN (nmol/mg Proteins)	MDA IN PMN (nmol/mg Proteins)	GSSG IN MN (nmol/mg Proteins)	GSSG IN PMN (nmol/mg Proteins)	GSH IN MN (umol/mg Proteins)	GSH IN PMN (umol/mg Proteins)	GSSG/GSH IN MN	GSSG/GSH IN PMN	OXY-SCORE
N° PMV	−0.187 (*p* = 0.152)	−0.025 (*p* = 0.859)	−0.214 (*p* = 0.202)	−0.285 (*p* = 0.177)	**−0.304** **(*p* = 0.076)**	0.267 (*p* = 0.139)	−0.13 (*p* = 0.329)	0.112 (*p* = 0.57)	−0.062 (*p* = 0.638)	−0.015 (*p* = 0.905)	−0.104 (*p* = 0.374)	−0.091 (*p* = 0.503)	−0.166 (*p* = 0.143)	0.012 (*p* = 0.94)	0.127 (*p* = 0.276)	−0.096 (*p* = 0.508)	−0.107 (*p* = 0.288)
CD31 IN PMV	0.081 (*p* = 0.548)	0.194 (*p* = 0.186)	0.136 (*p* = 0.41)	−0.304 (*p* = 0.149)	**−0.36 *** **(*p* = 0.047)**	−0.135 (*p* = 0.511)	**−0.35 *** **(*p* = 0.01)**	−0.159 (*p* = 0.403)	**−0.28 *** **(*p* = 0.046)**	−0.019 (*p* = 0.878)	−0.109 (*p* = 0.382)	−0.19 (*p* = 0.172)	−0.054 (*p* = 0.657)	0.046 (*p* = 0.76)	−0.053 (*p* = 0.669)	−0.32 (*p* = 0.831)	0.000 (*p* = 0.997)
CD41 IN PMV	**0.25** **(*p* = 0.056)**	0.216 (*p* = 0.12)	**0.32 *** **(*p* = 0.034)**	−0.321 (*p* = 0.102)	−0.191 (*p* = 0.28)	−0.064 (*p* = 0.743)	−0.193 (*p* = 0.156)	0.163 (*p* = 0.388)	−0.141 (*p* = 0.315)	0.032 (*p* = 0.797)	**−0.33 *** **(*p* = 0.005)**	0.036 (*p* = 0.791)	−0.094 (*p* = 0.42)	−0.201 (*p* = 0.16)	**−0.24 *** **(*p* = 0.04)**	**0.266** **(*p* = 0.059)**	0.098 (*p* = 0.351)
N° EMV	**−0.5 **** **(*p* = 0.000)**	**−0.31 *** **(*p* = 0.031)**	−0.272 (*p* = 0.114)	−0.123 (*p* = 0.596)	0.22 (*p* = 0.21)	−0.204 (*p* = 0.281)	−0.164 (*p* = 0.219)	−0.188 (*p* = 0.319)	−0.103 (*p* = 0.454)	0.132 (*p* = 0.293)	0.063 (*p* = 0.608)	**−0.24** **(*p* = 0.084)**	−0.044 (*p* = 0.713)	0.196 (*p* = 0.19)	0.049 (*p* = 0.689)	**−0.32 *** **(*p* = 0.025)**	−0.03 (*p* = 0.772)
CD31 IN EMV	−0.168 (*p* = 0.198)	−0.095 (*p* = 0.493)	0.042 (*p* = 0.79)	**−0.41 *** **(*p* = 0.027)**	0.062 (*p* = 0.729)	−0.158 (*p* = 0.413)	0.067 (*p* = 0.623)	−0.31 (*p* = 0.096)	−0.212 (*p* = 0.123)	−0.183 (*p* = 0.133)	−0.045 (*p* = 0.706)	−0.098 (*p* = 0.462)	0.128 (*p* = 0.266)	**0.246** **(*p* = 0.08)**	−0.139 (*p* = 0.237)	−0.16 (*p* = 0.257)	0.15 (*p* = 0.153)
N° PMV CD142+	**0.26 *** **(*p* = 0.045)**	**0.366 **** **(*p* = 0.009)**	0.11 (*p* = 0.531)	**0.385** **(*p* = 0.07)**	**−0.35 *** **(*p* = 0.028)**	0.093 (*p* = 0.613)	−0.156 (*p* = 0.225)	−0.061 (*p* = 0.73)	−0.002 (*p* = 0.991)	0.01 (*p* = 0.933)	−0.213 (*p* = 0.068)	0.019 (*p* = 0.889)	**−0.27*** **(*p* = 0.016)**	**−0.263** **(*p* = 0.07)**	0.093 (*p* = 0.425)	**0.25** **(*p* = 0.087)**	0.001 (*p* = 0.994)
CD142 IN PMV	**0.567 **** **(*p* = 0.000)**	**0.399 **** **(*p* = 0.007)**	0.024 (*p* = 0.891)	0.085 (*p* = 0.706)	**−0.370** ***** **(*p* = 0.048)**	−0.059 (*p* = 0.77)	**−0.50** ****** **(*p* = 0.001)**	−0.027 (*p* = 0.89)	0.075 (*p* = 0.627)	−0.077 (*p* = 0.55)	**−0.410** ****** **(*p* = 0.001)**	−0.152 (*p* = 0.292)	**−0.271** ***** **(*p* = 0.029)**	**−0.302** ***** **(*p* = 0.04)**	−0.126 (*p* = 0.332)	**0.272** **(*p* = 0.071)**	0.036 (*p* = 0.75)
EMV CD142+	0.011 (*p* = 0.933)	0.176 (*p* = 0.231)	0.075 (*p* = 0.663)	0.03 (*p* = 0.889)	−0.153 (*p* = 0.389)	−0.003 (*p* = 0.987)	−0.236 (*p* = 0.083)	0.067 (*p* = 0.731)	0.159 (*p* = 0.26)	0.019 (*p* = 0.885)	**−0.311** ****** **(*p* = 0.009)**	−0.140 (*p* = 0.316)	**−0.253** ***** **(*p* = 0.03)**	**−0.406** ****** **(*p* = 0.01)**	−0.032 (*p* = 0.793)	**0.350 *** **(*p* = 0.016)**	−0.028 (*p* = 0.792)
CD142 IN EMV	−0.014 (*p* = 0.945)	0.151 (*p* = 0.434)	−0.204 (*p* = 0.338)	−0.398 (*p* = 0.158)	0.223 (*p* = 0.296)	−0.289 (*p* = 0.192)	**−0.359** ***** **(*p* = 0.029)**	−0.317 (*p* = 0.162)	−0.11 (*p* = 0.557	0.195 (*p* = 0.199)	**−0.317** ***** **(*p* = 0.046)**	**−0.309** ****** **(*p* = 0.08)**	−0.076 (*p* = 0.621	−0.208 (*p* = 0.29)	−0.247 (*p* = 0.12)	0.044 (*p* = 0.823)	0.041 (*p* = 0.758)

XO, xanthine oxidase; GPx, glutathione peroxidase; MDA, malondialdehyde; CAT, catalase; SOD, superoxide dismutase; GSSG: Glutathione disulfide; GSH; Glutathione; MN, mononuclear leukocytes; PMN, polymorphonuclear leukocytes; PMVs, platelet microvesicles; EMVs, endothelial microvesicles. * *p* < 0.05; ** *p* < 0.01.
